# Trefoil Factor 3 (TFF3) Is Involved in Cell Migration for Skeletal Repair

**DOI:** 10.3390/ijms20174277

**Published:** 2019-09-01

**Authors:** Katharina Krüger, Sebastian Schmid, Friedrich Paulsen, Anita Ignatius, Patricia Klinger, Thilo Hotfiel, Bernd Swoboda, Kolja Gelse

**Affiliations:** 1 Department of Orthopaedic Trauma Surgery, University Hospital Erlangen, 91054 Erlangen, Germany; 2 Institute of Functional and Clinical Anatomy, University of Erlangen-Nuernberg, 91054 Erlangen, Germany; 3 Institute of Orthopaedic Research and Biomechanics, University of Ulm, 89081 Ulm, Germany; 4 Division of Orthopaedic Rheumatology, Department of Orthopaedics, University of Erlangen-Nuernberg, 91054 Erlangen, Germany

**Keywords:** TFF3, cell migration, chemotaxis, mesenchymal progenitor cells, fracture healing

## Abstract

The aim of the study was to explore the possible role of Trefoil Factor Family peptide 3 (TFF3) for skeletal repair. The expression of TFF3 was analyzed in human joint tissues as well as in a murine bone fracture model. Serum levels of TFF3 following a defined skeletal trauma in humans were determined by ELISA. The mRNA expression of TFF3 was analyzed under normoxia and hypoxia. Expression analysis after stimulation of human mesenchymal progenitor cells (MPCs) with TFF3 was performed by RT^2^ Profiler PCR Array. The effect of recombinant human (rh)TFF3 on MPCs was analysed by different migration and chemotaxis assays. The effect on cell motility was also visualized by fluorescence staining of F-Actin. TFF3 was absent in human articular cartilage, but strongly expressed in the subchondral bone and periosteum of adult joints. Strong TFF3 immunoreactivity was also detected in murine fracture callus. Serum levels of TFF3 were significantly increased after skeletal trauma in humans. Expression analysis demonstrated that rhTFF3 significantly decreased mRNA of ROCK1. Wound healing assays showed increased cell migration of MPCs by rhTFF3. The F-Actin cytoskeleton was markedly influenced by rhTFF3. Cell proliferation was not increased by rhTFF3. The data demonstrate elevated expression of TFF3 after skeletal trauma. The stimulatory effects on cell motility and migration of MPCs suggest a role of TFF3 in skeletal repair.

## 1. Introduction

Trefoil Factor family peptide 3 (TFF3) is a 6.5 kDa peptide that is released after injury in a number of tissues and that is involved in tissue repair. TFF3 is characterized by a trefoil motif containing three conserved disulfide bonds and predominantly exists as a high molecular weight heteromer [1,2,3]. Its biological role has been intensively studied in gastrointestinal tissue, where it is stored in goblet cells. Beside the gastrointestinal tract, human TFF3 has been found in nearly all mucosae and several glands, but also in non-epithelial structures such as skeletal tissue including articular cartilage and bone [4,5]. The functions of TFF3 are various. It is supposed to interact directly with mucins by increasing viscosity and elasticity of mucin containing fluids like gastrointestinal or respiratory mucus or the tear film [2,6]. For long, it has been hypothesized that this may be their leading mechanism of action. However, in the meantime, it has also been shown that motogenic effects of TFF3 play an important role during epithelial restitution following epithelial injury and ensure rapid sealing of the epithelial layer after injuries [1,6]. For a long time, receptors for TFFs have been unknown. In 2009, the chemokine receptor type 4 (CXCR4, also known as stromal cell-derived factor 1 [SDF-1] receptor) was described as a low affinity receptor for TFF2 [7], and just recently it was shown that dimers of CXCR4 and CXCR7 are involved in TFF3-dependent activation of cell migration, but not cell proliferation [8,9]. By promoting motility of epithelial cells, TFF3 is involved in the maintenance and repair of mucosa. This has also been demonstrated for the avascular cornea, where TFF3 is expressed in the superficial epithelial layer of healthy human cornea and is strongly upregulated under inflammatory conditions supporting re-epithelialization of corneal wounds [10,11,12]. Articular cartilage is another avascular tissue that is characterized by lack of TFF3 in its healthy intact status, but upregulated TFF3 expression was found in osteoarthritic cartilage [5]. Stimulation of cultivated articular chondrocytes with recombinant (r) human (h) TFF3 resulted in a significantly increased production of cartilage-degrading matrix-metalloproteinases (MMPs) [5]. In mouse fetal epiphyses, TFF3 was not detected within the resting cartilage, but was present in cartilage undergoing endochondral ossification [4]. 

According to the current knowledge, a putative role for TFF3 after skeletal trauma can be hypothesized. Therefore, the aim of this study was to examine the presence and effects of TFF3 in skeletal tissue. Therefore, we first investigated the expression of TFF3 in human healthy joint tissues as well as in a murine fracture model. Furthermore, we explored the function of TFF3 on mesenchymal progenitor cells (MPCs) by assays focusing on cell migration, cell motility, and proliferation.

## 2. Results

### 2.1. Expression of TFF3 in Human Joint Tissue

**Figure 1:**
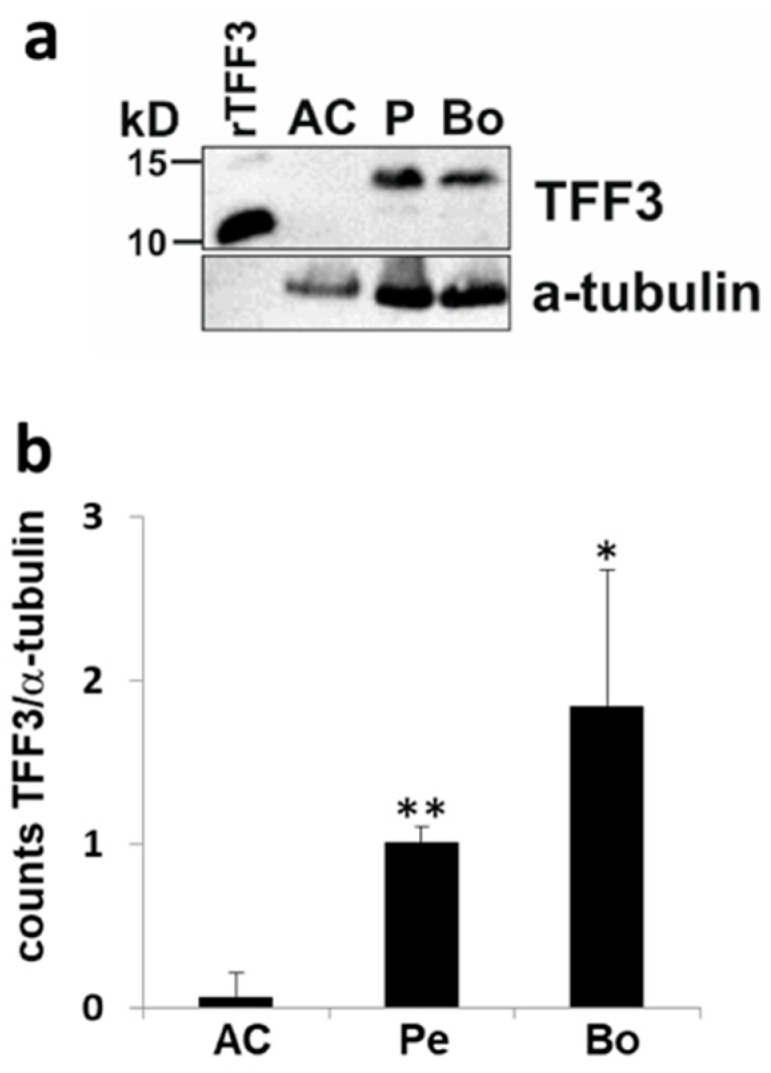
Expression of Trefoil Factor Family peptide 3 (TFF3) in human osteochondral tissue. Western blot analysis of TFF3 peptide in human adult knee joint tissue including articular cartilage (AC), periosteum (P), and bone (Bo) (**a**). Band intensities of TFF3 from *n* = 3 different donors were quantified by measuring the integral optical density (**b**). * *p* < 0.01; ** *p* < 0.001.

TFF3 protein could not be detected in intact human adult articular cartilage by immunoblotting, but abundantly in human adult periosteal tissue and subchondral bone (Figure 1a). The quantification of the TFF3 protein bands by integral optical density revealed significantly higher TFF3 levels in periosteal tissue (*p* < 0.001) and bone (*p* = 0.02) compared to articular cartilage (Figure 1b).

### 2.2. Expression of TFF3 in Murine Fracture Callus

**Figure 2:**
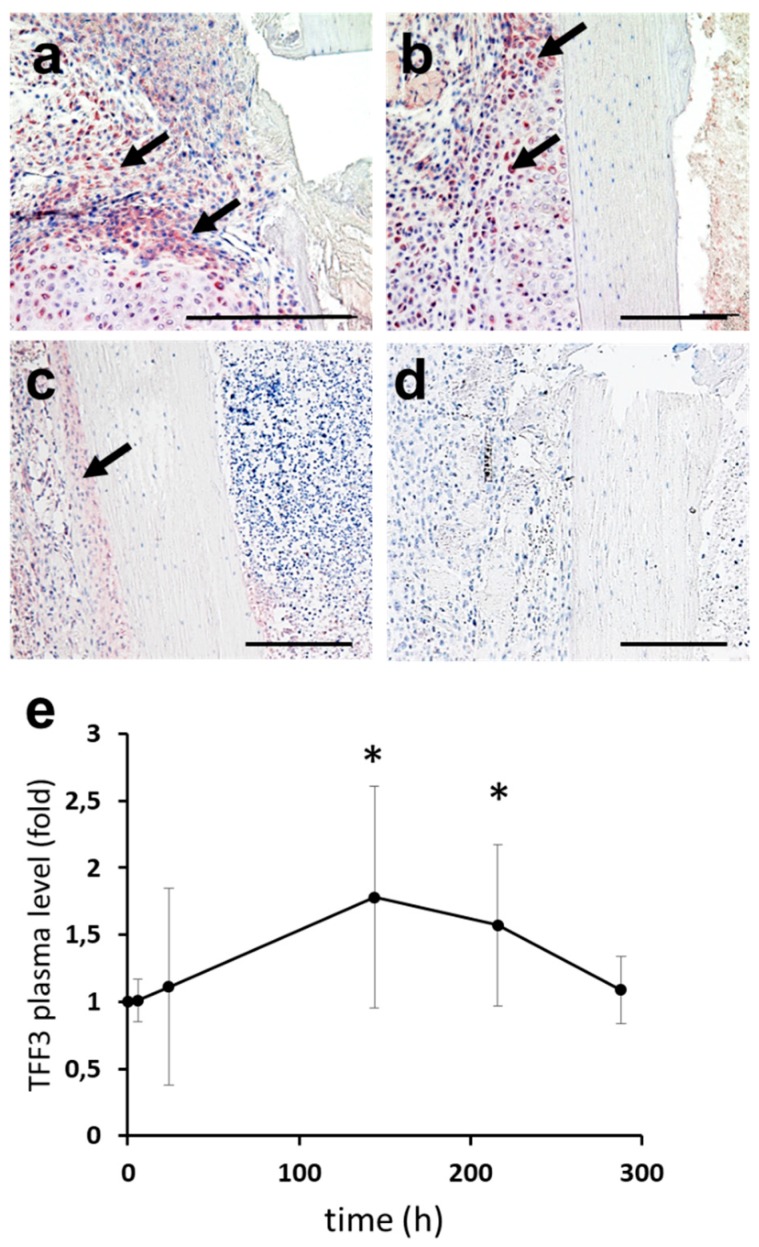
Expression of TFF3 under pathological conditions. Immunoreactivity for TFF3 10 days following defined fracture of the femur in a murine model. Positive TFF3 reactivity in soft callus (**a**) and cartilaginous callus (**b**) (arrows). Osteocytes of cortical bone and hematopoietic cells within bone marrow in healthy areas largely lack TFF3 reactivity, whereas periosteal cells are characterized by distinct TFF3 immunoreactivity (arrow) (**c**). Negative control (**d**). ELISA analysis reveals a significant increase of serum TFF3 at six and nine days following total knee replacement (**e**). The graphs show the mean ± SD expression levels of TFF3 (normalized to β2-microglobulin) in samples from three different donors. * *p* < 0.05. bars = 100 µm.

Fracture callus tissue was mainly composed of fibrous and cartilaginous tissue at day 10 post-surgery (Figure 2a,b). Most fibroblasts and chondrocytes of the callus strongly reacted positive for TFF3 (Figure 2a,b). Osteocytes of cortical, lamellar bone, and hematopoietic cells within bone marrow in healthy areas in some distance to the osteotomy hardly reacted with the TFF3 antiserum, whereas periosteal cells were characterized by positive TFF3 reactivity (Figure 2c). No staining was observed in negative controls (Figure 2d).

### 2.3. Upstream Regulators of TFF3: Induction of TFF3 by Trauma 

In order to analyze the temporal release of TFF3 following a defined skeletal trauma, serum was obtained from patients who underwent total knee replacement. The osteotomy by an oscillating saw across a large bony area represents a defined trauma, which is more reproducible than rather undefined clinical fracture situations. A transient increase in TFF3 serum levels could be detected with significantly increased levels between six days (*p* = 0.02) and nine days (*p* = 0.04) post-trauma (Figure 2e).

### 2.4. Effects of TFF3 on Cell Migration and Proliferation of MPCs

**Figure 3:**
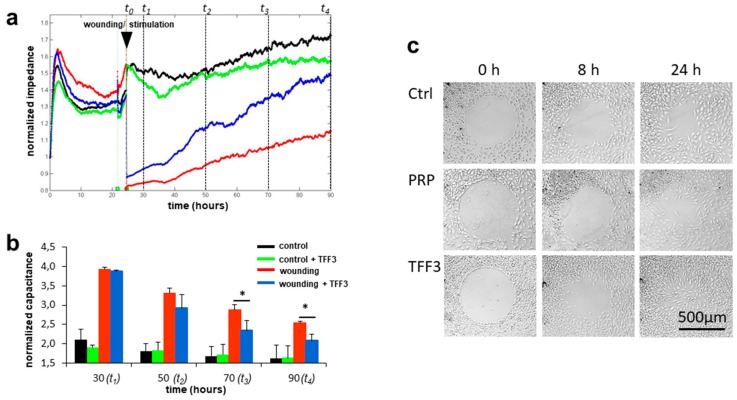
Influence of TFF3 on cell migration. Electric cell-substrate impedance sensing (ECIS) analyzed the 2D-cell migration over a 90 h period following electrical wounding or in non-wounded controls in the presence or absence of rhTFF3. A representative time-impedance diagram with the relevant time points for statistical analysis is shown in (**a**). Increased cell migration into wounded areas correlated inversely with electrical resistance (**b**). Radius migration assay confirms rapid and even ingrowth of cells stimulated by TFF3 (**c**). Bars show the mean ± SD. * *p* < 0.05.

In order to evaluate the effect of TFF3 on wound healing of MPCs cultivated in monolayer conditions, an automated migration assay based on the ECIS method was performed. The wounding-induced defect of the cell monolayer is gradually recovered by adjoining cells migrating into the defect. An increasing cell coverage of the wounded area increases the electrical impedance, or reduces the capacitance, respectively. Without wounding, rhTFF3 had no significant effect on the cell viability on a confluent cell monolayer (Figure 3a). However, after wounding, the presence of 10 µg/mL rhTFF3 resulted in an increased ingrowth of MPCs into the defect area, which was significant at 50 (t_2_), 70 (t_3_), and 90 h (t_4_) after cell seeding, which corresponds to 25.5, 45.5, and 65.5 h after wounding, respectively (Figure 3b).

### 2.5. Radius Cell Migration Assay

The two-dimensional migration of MPCs into a cell-free spot was visualized by the radius cell migration assay. Microscopic images were taken at different time points. The medium either contained 20 µL/mL PRP or 10 µg/mL rhTFF3. Medium without growth factors served as a control. In all three cases, the cells started to migrate as soon as 8 h (Figure 3c). At 24 h, rhTFF3 markedly enhanced the ingrowth of cells into the cell-free area with the cells spreading all over the former cell-free spot. This was clearly different to non-stimulated cells, which had only invaded the outer zone of the clear space in this interval. The application of PRP resulted in localized cellular nodule formation, but the cells failed to evenly spread all over the clear space at 24 h (Figure 3c).

### 2.6. Cell Proliferation and Cell Viability 

**Figure 4:**
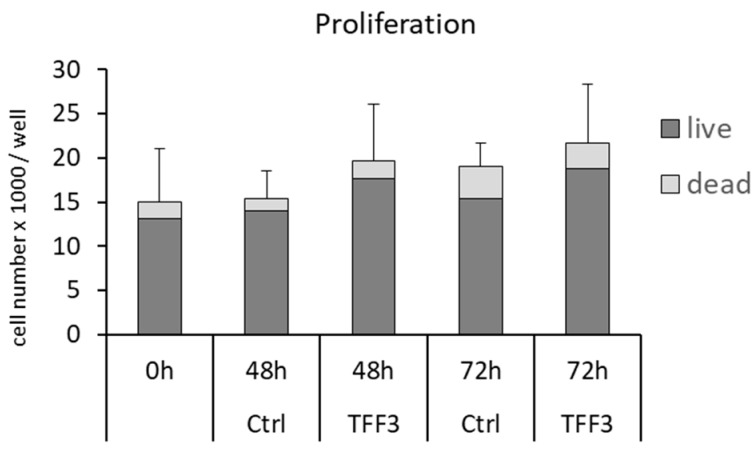
Influence of TFF3 on cell proliferation and viability. Neither proliferation nor viability of mesenchymal progenitor cells (MPCs) is influenced by 10 µg/mL rhTFF3. Bars show the mean ± SD.

A cell proliferation assay in monolayer cultures analyzed whether the detected ingrowth of MPCs into wounded areas (ECIS) may also result from increased cell proliferation. However, the application of 10 µg/mL rhTFF3 had no significant effect on the proliferation of MPCs at t = 48 h or t = 72 h (Figure 4). The application of rhTTF3 did not influence the number of dead cells in this assay.

### 2.7. Chemotaxis Assay

**Figure 5:**
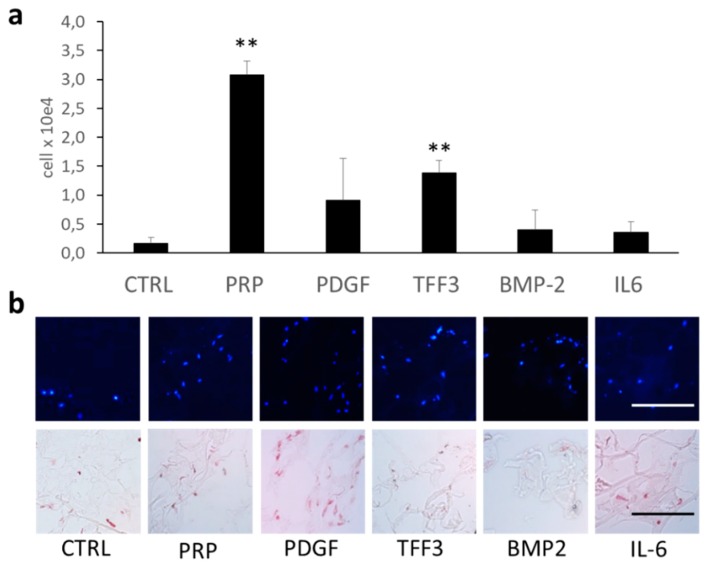
Chemotaxis assay. The chemotactic effect of a number of growth factors and cytokines was detected by 3-dimensional ingrowths of MPCs into a collagen gel. Platelet-rich plasma concentrate (PRP) and TFF3 significantly stimulated the ingrowth of MPCs, while other growth factors and cytokines had minor effects (**a**). DAPI and Fast Red staining visualize the ingrown cells within the three-dimensional collagen matrix (**b**). Bars = 100 µm. ** *p* < 0.01.

The invasion of repair cells (MPCs) into a three-dimensional matrix was analyzed by an assay consisting of a cellular monolayer and overlying cell-free collagen-hydrogel loaded with different chemotactic agents including PRP, PDGF, TFF3, BMP-2, and IL-6 (Figure 5a,b). The collagen hydrogel without any chemotactic agent (control) did hardly attract any cells from the underlying monolayer. In contrast, PRP and rhTFF3 significantly supported the invasion of cells into the three-dimensional matrix. The application of PDGF also increased the number of cells within the matrix, which, however, did not reach the level of significance. BMP-2 and IL-6 only had minor chemotactic effects on MPCs.

### 2.8. Immunofluorescence

**Figure 6:**
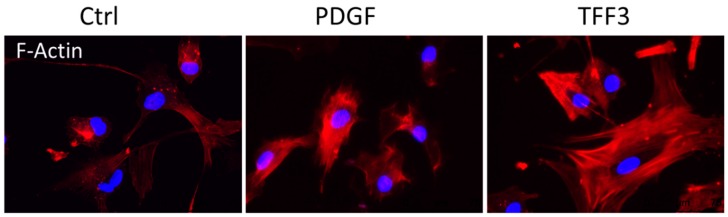
Effect on F-Actin cytoskeleton. Conjugated phalloidin visualizes changes on the F-Actin cytoskeleton. Stimulation by Platelet derived Growth Factor (PDGF) and TFF3 lead to reorganized F-Actin with more prominent stress fibers compared to control.

The ability of Phalloidin to bind to F-Actin served to visualize the effects of TFF3 on the cytoskeleton that are responsible for motility of MPCs (Figure 6).

The application of TFF3 markedly influenced the recruitment and reorganization of the F-Actin cytoskeleton. TFF3 increased the number of stress fibers and lamellipodia compared to non-stimulated control cells. PDGF also supported the formation of the F-actin cytoskeleton with distinct stress fibers, but less impressive lamellipodia compared to TFF3.

### 2.9. RT^2^ Profiler PCR Array Analysis

The analysis of the expression of 84 genes involved in cell motility revealed significant downregulation of *ROCK1* (*p* = 0.043) by TFF3 compared to control cells (Table 1). The impact on the expression of other genes did not reach the level of significance using this array.

## 3. Discussion

Our data highlight the role of TFF3 for chemotaxis and migration of mesenchymal progenitor cells (MPCs), which implicates a function in repair and remodeling of skeletal tissues. 

We could detect increased TFF3 reactivity within the soft tissue and the cartilaginous fracture callus in a murine model. Furthermore, we measured increased serum levels of TFF3 in patients in the first days after total knee replacement, which is a procedure that is associated with a defined osseous trauma by osteotomy of the subchondral bone. Indeed, bone trauma elicits a defined acute phase response with a multitude of molecular mechanisms [13].

One central biological role of the early inflammation phase during tissue repair is the recruitment of stem or progenitor cells and their guidance by chemotaxis to the site of injury. Indeed, the important role of TFF3 for chemotaxis and migration of repair cells has been demonstrated in several tissues including mucosal tissue and the cornea [1,10,14,15]. The data of the current study demonstrate that these properties of TFF3 can also be transferred to the skeletal system. We could confirm a distinct effect of rhTFF3 on the ingrowth of MPCs into wounded areas using two-dimensional systems such as the ECIS wound healing assay or three-dimensional assays based on a collagen hydrogel. The stimulatory effect of TFF3 on wound healing can predominantly be ascribed to increased cell migration, since we could not detect significant effects of TFF3 on cell proliferation or cell viability. 

Chemotaxis is based on a concentration gradient of bioactive factors. Thus, it is evident that the local concentration of the attractant needs to be much higher than the level within the surrounding tissue or serum. According to the literature and our ELISA data, the serum levels of TFF3 range from 1 to 20 ng/mL [16,17]. Therefore, it is plausible that the concentration of the spatially restricted chemotactic agent needs to be much higher, e.g., 10 µg/mL, as used in this study. Indeed, it is very difficult to quantify such peak concentrations that occur highly spatially restricted within the tissue by conventional methods (e.g., ELISA).

According to the current knowledge, the stimulatory effect of TFF3 on the motility of epithelial and mesenchymal cells involve a number of mechanisms including the cytoskeleton, cell–cell adhesions, and cell-substrate contacts [15,17]. In the present study, we could demonstrate distinct effects of TFF3 on the organization of the F-Actin cytoskeleton with formation of prominent stress fibers and lamellipodia. Recent studies showed that TFF3 acts via a number of different pathways [18]. In MPCs, we could show that TFF3 significantly decreases the mRNA expression of *ROCK1*. Recently, ROCK1 was shown to function as a suppressor of cell migration by stabilizing PTEN. Thus, downregulation of *ROCK1* activates downstream targets of PTEN including PIP3, AKT, GSK-3β, and cyclinD1 [19]. Indeed, the PI3K/AKT signaling pathway was shown to mediate the effects of TFF3 on cell migration [20,21]. Other studies demonstrated that enhanced cell motility induced by TFF3 is also mediated via the mitogen-activated protein kinases (MAPKs) c-Jun N-terminal kinase (JNK), p38, and Ras/MEK/ERK, as well as via the JAK/STAT3 pathway [18,21,22,23,24,25]. Another mechanism of TFF3 for stimulating cell motility involves the internalization or degradation of E-cadherin, which reduces cell–cell contacts necessary for cell migration [15]. Degradation of E-cadherin can be mediated by MMP2 and MMP9, which are also targets of TFF3 [24,26]. Furthermore, it was demonstrated that CXCR4 and CXCR7 act as receptors for TFF3, which results in a pathway independent from the MAPK signaling cascade [8,9].

To date, there are only limited data on the upstream regulators of TFF3. Inflammation is an important upstream regulator of TFF3. In particular, inflammatory cytokines including IL-1 and TNFα were shown to induce gene expression and activity of TFF3 [5]. Indeed, skeletal tissue trauma includes an early inflammatory phase, which may trigger TFF3 activity. To date, it might be speculated whether other factors, such as mechanical stress, contribute to the induction of TFF3. Characterization of the TFF3 gene promotor revealed a SP1 binding site [27]. Since the transcription factor SP1 is thought to be involved in stress-induced induction of a number of genes [28,29], it may provide a link to mechanical TFF3-induction also in osteochondral tissue.

In conclusion, our data outline a role of TFF3 in the repair of skeletal tissue following trauma. Tissue trauma seems to induce TFF3 activity. For mesenchymal progenitor cells, TFF3 markedly supports cell motility and acts as a chemotactic agent. Since cell migration is an integral part of any healing process, future studies will have to explore the effect of exogenously applied rhTFF3 on non-union fractures, cartilage lesions, and other repair processes of the skeletal system.

## 4. Methods

### 4.1. Human Tissue Samples

Human adult articular cartilage (AC), periosteal mesenchyme (P), and subchondral bone (Bo) were isolated from respective knee joints of 10 patients (mean age 67.0 years, range 54–84 years) undergoing total knee arthroplasty for osteoarthritis at the Department of Orthopedic Trauma Surgery and the Division of Orthopedic Rheumatology at the University of Erlangen-Nuernberg. The diagnosis of primary osteoarthritis was based on clinical and radiographic evaluations according to the standard criteria [30]. Secondary osteoarthritis or rheumatoid arthritis was excluded. Macroscopically intact articular cartilage was isolated from osteochondral specimen originating from the resected dorsal femoral condyles with a macroscopically intact joint surface without apparent fissures characterized by an Outerbridge score of 0 or 1. Periosteal mesenchyme and subchondral bone were isolated from osteochondral samples of the same respective joints. Each patient gave informed consent prior to surgery, and the institutional ethics committee approved the study (Ref. No. 3555; Date of Approval: 2017-02-17; Ethics committee of the University of Erlangen-Nuernberg).

### 4.2. Immunoblot Analysis

To obtain whole cell protein extracts, the cells of minced articular cartilage, periosteal tissue, and minced subchondral bone, were lysed in Laemmli buffer (2% sodiumdodecylsulfate (SDS) and 60 mM Tris (pH 6.8)) in the presence of a protease inhibitor mix (1:200) (Sigma-Aldrich, Taufkirchen, Germany), homogenized by sonification, heated for 5 min at 95 °C, and centrifuged at 16,000 g for 15 min at 4 °C. Supernatants were used to detect TFF3 peptide levels. Total peptide concentrations of the extracts were quantified using the BCA protein assay kit (Pierce, Rockford, IL, USA). Western blotting was performed according to a recently described protocol [31].

For statistical analysis, the integrated optical density (IOD) of each band for TFF3 and α-tubulin immunoblots was measured with an image analysis program that uses 2-dimensional densitometry Image Studio Lite Version 5.2 (LICOR, Lincoln, NE, USA). The IOD value of each TFF3 band was normalized to the value of the respective band of the α-tubulin immunoblot.

### 4.3. Murine Fracture Callus

TFF3 expression during bone fracture healing was analyzed in a standardized murine femur fracture model that has been described previously in detail [32,33]. The animal experiment was in compliance with international regulations for the care and use of laboratory animals (Directive 2010/63/EU) with the approval of the Local Ethical Committee (No. 1079, Regierungspräsidium Tübingen, Germany). Briefly, a 0.5 mm osteotomy gap was created in the femur of female 36-week old C57BL/6 mice and stabilized by an external fixator. The fracture calli were harvested from 3 mice killed 10 days post-surgery, a time point of extensive endochondral bone formation, fixed in 4% paraformaldehyde for 12 h, followed by decalcification in 0.5 M EDTA for 4 weeks and embedded in paraffin after standard processing.

### 4.4. Immunohistochemical Analysis

Murine femoral fracture sections were used for histological analysis. After standard processing, serial transverse 5-µm sections were cut and stained with hematoxylin/eosin for histological assessment. For immunohistochemistry with an antiserum against TFF3 [34], deparaffinized sections were pretreated with 0.05% trypsin for 60 min. The sections were then left to react with anti-human TFF3, serum (anti-rTFF3-1) [34] diluted 1:300, followed by incubation with biotinylated secondary antibodies (Dianova, Hamburg, Germany). Negative control sections were incubated with isotype normal mouse IgG (Santa Cruz Biotechnology). Bound antibodies were visualized by exposure to a complex of avidin and biotinylated peroxidase H (Vectastain-Elite-ABC Kit, Vector Laboratories, Burlingame, CA). The sections were developed with AEC (3-amino-9-ethylcarbazole) (DAKO, Hamburg, Germany) and counterstained with hematoxylin.

### 4.5. Human Serum Samples

After approval of the institutional ethics committee (Ref.No. 3555; University of Erlangen-Nuernberg), blood sampling was done in 10 patients (mean age 67.0 ± 10.1 years, range 54–84) undergoing total knee arthroplasty directly preoperatively (t = 0), as well as 6 h, 24 h (1 day), 144 h (6 days), 216 h (9 days), and 288 h (12 days) postoperatively. Serum TFF3 levels were analyzed by using a commercially available sandwich-enzyme-linked immunosorbent assay Kit according to the manufacturer’s protocol (human TFF3 Quantikine ELISA Kit, R&D Systems, Minneapolis, MN, USA).

### 4.6. Cell Culture Experiments

Mesenchymal progenitor cells (MPCs) were isolated from the periosteal/synovial rim at the border of the joint surface of resected femoral condyles from above mentioned donors undergoing total knee arthroplasty. 

The tissue was minced with a scalpel, and cells were released by treatment with 2 mg/mL Pronase in Hanks’ balanced salt solution for 30 min at 37 °C, followed by exposure to 180 units/mL collagenase in DMEM (Sigma-Aldrich, Hamburg, Germany) supplemented with 10% fetal calf serum (FCS) and 1% penicillin/streptomycin (P/S) and 0.1% amphotericin for 16 h at 37 °C. After digestion, cells were cultivated in DMEM supplemented with 10% FCS, 1% P/S, and 0.1% amphotericin for about 7 days and then used for stimulation experiments.

### 4.7. RT^2^ Profiler PCR Array of Human Cell Motility

MPCs from 4 human adult donors were independently seeded at a density of 1 × 10^5^ cells/cm^2^ in 24 well-plates for the following stimulation experiments. For adequate stimulation by rhTFF3, we refer to the extensive dose-finding experiments by Diekow et al. who found a ceiling-effect for concentrations exceeding 10 µg/mL [8]. Therefore, we chose this concentration of rhTFF3 for stimulatory experiments. After 24 h, the medium was changed to serum-free medium for 3 h, followed by stimulation with 10 µg/mL rhTTF3 (ImmunoTools, Friesoythe, Germany) in DMEM supplemented with 1% P/S, and 1× insulin-transferrin-selenium (1× ITS)(Sigma-Aldrich, Hamburg, Germany) for 24 h. RNA extraction from stimulated cells and non-stimulated control cells was performed using the High Pure RNA Isolation Kit (Roche, Mannheim, Germany) according to the manufacturer’s protocol. The quality of RNA was analyzed using a 2100 Bioanalyzer system (Agilent Technologies, Santa Clara, CA, USA). RT^2^ First Strand kit (Quiagen, Hilden, Germany) was used for reverse transcription. RT^2^ SYBR Green/ROX qPCR Master Mix was used for quantitative PCR on Qiagen RT^2^ Profiler PCR Array of human cell motility. The RT-PCR Array was performed on 7500 Sequence Detection System (Applied Biosystems, Foster City, CA, USA), including all control reactions recommended by the arrays’ manufacturer. Target gene expression level was calculated according to recommendations of the Qiagen RT^2^ Profiler PCR Array, applying the comparative threshold cycle (CT) method, with glyceraldehyde-3-phosphate dehydrogenase (GAPDH) as a reference gene.

### 4.8. Electric Cell-Substrate Impedance Sensing (ECIS)

Wound healing was analyzed by the electric cell-substrate impedance sensing (ECIS) instrument *Z theta+16* (Applied Biophysics, New York, USA) using ECIS culture ware *8W2LE* arrays. Array slides were incubated with DMEM, 10% FCS, and 1% P/S overnight before cells (MPCs) were seeded at a density of 60,000 cells per cavity. The adhesion of cells was tracked after 1.5 h, followed by a proliferation phase of additional 20 h in DMEM supplemented with 10% FCS. After change to serum-free medium for another 3 h, the cell monolayers were electrically wounded with 2400 µA at 60,000 Hz for 30 s. Cells were then stimulated with 10 µg/mL rhTTF3 in DMEM, 1% penicillin/streptomycin, and 1 × ITS. Impedance and capacitance of the cell layer were recorded for a further culture period of 3 days by multi-frequency analysis using the *ECIS Z theta* software. All experiments were repeated independently with cells from three different donors.

### 4.9. Radius Cell Migration Assay

The Radius™-Cell-Migration Assay Kit (Cell Biolabs, San Diego, CA, USA) utilizes a 24-well plate to monitor the two-dimensional migratory properties of MPCs. Each well contains a 0.68 mm biocompatible hydrogel spot where cells cannot attach. For this experiment, 5 × 10^4^ MPCs were seeded into the Radius™-Cell-Migration well and attached outside of the gel-coated area. After 24 h of culturing, the hydrogel was removed to expose a cell-free region to study cell migration with subsequent closure of the cell-free space. The cells were stimulated either by 10 µg/mL rhTFF3 or 20 µl/mL platelet-rich plasma concentrate (PRP) in DMEM supplemented with 10% FCS and 1% penicillin-streptomycin to yield a total volume of 1 ml per well. PRP was prepared as described previously [35]. The ingrowth of cells was compared to negative controls at defined time points. Microscopic images were performed in bright field (Axiophot (HBO100), Zeiss, Jena, Germany) at 0 h, 8 h, 24 h, and 48 h (not shown) to document cell migration.

### 4.10. Proliferation Assay

MPCs from three different donors were seeded at 2.5 × 10^4^ cells per well (1.9 cm^2^) in 24-well plates and pre-cultivated in DMEM (1× ITS, 1× P/S) for 24 h to allow cell attachment and to yield a semi-confluent cell density. After this pre-cultivation period, non-adherent cells were removed and the remaining cell layer was cultured in DMEM (1× ITS, 1% P/S) supplemented with or without 10 µg/mL rhTFF3. At t = 0 h, t = 48 h, or t = 72 h, the cells of separately cultivated wells were stained with trypan blue and released from the culture plates to quantify live and dead cells using a hemocytometer.

### 4.11. Chemotaxis Assay

To imitate the invasion of repair cells into a three-dimensional matrix upon chemotactic stimuli, we utilized a collagen type I-hydrogel (Chondrofiller liquid, Amedrix GmbH, Esslingen, Germany) into which different chemotactic factors were applied. Cells from an underlying monolayer cell sheet were attracted into the overlying collagenous matrix. The number of invading cells was quantified by histology and cell counting. 

In detail, MPCs were seeded into silicon chambers (2-well Silicone chamber, Ibidi, Martinsried, Germany) (2 × 10^4^ cells per 0.22 cm^2^ silicone chamber) and were allowed to adhere and form a monolayer in DMEM with 2% FCS and 1% penicillin-streptomycin. After 24 h, the cell monolayer was washed to remove non-attached cells, followed by application of 100 µL of collagen type I-hydrogel (Chondrofiller liquid) onto the cell monolayer in each silicon chamber. After solidification of the gel, a volume 20 µL of the respective chemotactic factors, including rhTFF3 (10 µg/mL, R&D Systems, Minneapolis, MN, USA), PRP concentrate, PDGF (Platelet derived Growth Factor, 100 ng/ mL, R&D Systems), TGF-β 1 (transforming growth factor β1, 100 ng/mL, R&D Systems), BMP-2 (bone morphogenetic protein 2, 100 ng/mL, R&D Systems), and IL-6 (Interleukine 6, 100 ng/mL, Immunotools, Friesoythe, Germany), was applied onto the hydrogel in the given concentrations, followed by application of another 100 µL collagen type-I hydrogel. After solidification of the gel, the chambers were transferred into 6-well plates and cultivated in DMEM supplemented with 2% FCS and 1% penicllin-streptomycin. Hydrogels without chemotactic factors served as control samples. 

After 14 days, the collagen matrices were harvested from the silicon chambers and thoroughly washed with PBS to remove externally attached cells. The matrices were digested with 0.1% clostridial collagenase (Roche, Mannheim, Germany) for 45 min at 37 °C in order to release invaded cells from the matrix. The total number of released cells that had invaded the matrix were quantified using a Neubauer counting chamber. Alternatively, the remaining samples were fixed in 4% paraformaldehyde (PFA) and embedded with Tissue-tek (Sakura, Staufen, Germany) for frozen sections. The cells within the matrix were visualized with DAPI (Roth, Karlsruhe, Germany) and Nuclear Fast Red (Merck Millipore, Darmstadt, Germany). All experiments were performed with triplicates.

### 4.12. Immunofluorescence

MPCs (5 × 10³ cells) were seeded on round chamber slides and cultured for 24 h in DMEM without further stimulus, or in medium containing either a final concentration of 10 µg/mL TFF3 or 100 ng/mL PDGF. The cells were fixed with 4% PFA for 10 min followed by thorough washing with PBS. The permeability of the cells was increased by adding 0.1% TritonX-100 for 3 min, followed by washing with PBS. The cells were exposed to Phalloidin conjugate solution (Alexa Fluor 568 Phalloidin; Thermo Fisher) at room temperature for 60 min to visualize the F-Action cytoskeleton. After washing with Tris buffered saline (TBS; 5 mM Tris in 0.9% NaCl, pH 7.35) the cells were covered with a mounting medium containing 4´-6´-Diamidino-2-phenylindole (Vector Inc., Peterborough, UK). The F-Actin cytoskeleton was investigated under a fluorescence microscope.

### 4.13. Statistical Analysis

All data are presented as mean ± SD. Serum levels of TFF3 and the quantification of chemotaxis were analyzed by one-way analysis of variance (ANOVA) followed by Dunnett’s multiple comparison test. Quantitative gene expression and cell proliferation were analyzed using Student’s two-sided *t*-test. The measurement of the different capacitances by the ECIS-method was determined using the pair-matched one-way ANOVA test followed by the Tukey multiple comparison test. Statistical analysis was done by GraphPadPrism6 software V 6.04. All statistical results were considered significant for *p*-values < 0.05.

## Figures and Tables

**Table 1:** Differentially expressed genes upon rhTFF3 stimulus (RT^2^ Profiler PCR Array Human Cell Motility). * p < 0.05.

Gene	Fold Change	*p*-Value
*CAPN1*	0.630	0.151
*ARHGDIA*	0.793	0.109
*STAT3*	0.851	0.219
*BAIAP2*	0.866	0.443
*ROCK1*	0.868	0.043 *
*SH3PXD2A*	0.869	0.456
*PLD1*	0.871	0.562
*IGF1*	0.872	0.690
*BCAR1*	0.874	0.499
*PRKCA*	0.879	0.478
*PTK2*	0.881	0.073
*PTEN*	0.882	0.499
*ACTN3*	1.091	0.554
*ACTB*	1.101	0.567
*MMP9*	1.145	0.916
*EGF*	1.155	0.991
